# *Toxoplasma gondii* in domiciled dogs and cats in urban areas of Brazil: risk factors and spatial distribution

**DOI:** 10.1051/parasite/2021049

**Published:** 2021-07-08

**Authors:** Igor Falco Arruda, Patricia Riddell Millar, Alynne da Silva Barbosa, Luiz Claudio de Souza Abboud, Izabel Cristina dos Reis, Alex Sander da Cruz Moreira, Mariana Pedrosa de Paula Guimarães, Maria Regina Reis Amendoeira

**Affiliations:** 1 Laboratório de Toxoplasmose e outras Protozooses, Instituto Oswaldo Cruz/Fiocruz Av. Brasil, 4365-Manguinhos 21040-900 Rio de Janeiro Brazil; 2 Departamento de Microbiologia e Parasitologia, Instituto Biomédico/Universidade Federal Fluminense Rua Professor Hernani Melo, 101/212a-São Domingos 24210-130 Niterói Brazil; 3 Instituto Municipal de Medicina Veterinária Jorge Vaitsman, SUBVISA/Secretaria Municipal de Saúde Av. Bartolomeu de Gusmão, 1120-São Cristóvão 20941-160 Rio de Janeiro Brazil; 4 Laboratório de Mosquitos Transmissores de Hematozoários, Instituto Oswaldo Cruz/Fiocruz Av. Brasil, 4365-Manguinhos 21040-900 Rio de Janeiro Brazil

**Keywords:** Toxoplasmosis, Companion animals, Serology, Rio de Janeiro metropolitan region

## Abstract

*Toxoplasma gondii* is a highly prevalent zoonotic parasite in Brazil capable of infecting mammals and birds. The increase in the urban populations of pets and the narrowing of the human–animal relationship can facilitate the transmission of important public health zoonoses, such as toxoplasmosis. This study aimed to evaluate the frequency and spatial distribution of *T. gondii* infection and its risk factors in domiciled dogs and cats attended at the Jorge Vaitsman Institute, Rio de Janeiro. Serum samples from 400 dogs and 272 cats were evaluated by an indirect fluorescent antibody test (IFAT) for IgG anti-*T. gondii* antibodies. Epidemiological questionnaires were used to interview the animals’ owners to identify risk factors for infection. Of the total, 34% (136/400) of dogs and 8.1% (22/272) of cats had anti-*T. gondii* antibodies. Breed (OR: 2.10–95%, CI 1.27–3.46) was a risk factor for dogs, while sex (OR: 3.40–95%, CI 1.10–10.52) and homemade food consumption (OR: 8.49–95%, CI 2.48–29.05) were risk factors for cats. Offal consumption was considered a risk factor for both species evaluated (OR: 2.74–95%, CI 1.38–5.43 for dogs; OR: 7.66–95%, CI 1.24–47.29 for cats). The spatial analysis showed that *T. gondii* seropositive animals were widely distributed in the metropolitan region of Rio de Janeiro state, with a concentration observed mainly in the west and north zones of Rio de Janeiro city. The results emphasize the importance of adopting prophylactic measures to control *T. gondii* transmission in domiciled dogs and cats in Rio de Janeiro, contributing positively to public health.

## Abstract (fr)

*Toxoplasma gondii* est un parasite zoonotique très répandu au Brésil, capable d’infecter les mammifères et les oiseaux. L’augmentation des populations urbaines d’animaux de compagnie et le rétrécissement de la relation homme-animal peuvent faciliter la transmission de zoonoses importantes pour la santé publique, telles que la toxoplasmose. Cette étude visait à évaluer la fréquence et la distribution spatiale de l’infection à *T. gondii* et ses facteurs de risque chez les chiens et les chats domiciliés qui ont fréquenté l’Institut Jorge Vaitsman de Rio de Janeiro. Des échantillons de sérum de 400 chiens et 272 chats ont été évalués par un test d’immunofluorescence indirecte (IFAT) pour les anticorps IgG anti-*T. gondii*. Des questionnaires épidémiologiques ont été appliqués aux propriétaires des animaux pour identifier les facteurs de risque d’infection. Sur le total, 34 % (136/400) des chiens et 8,1 % (22/272) des chats avaient des anticorps anti-*T. gondii*. La race (OR : 2,10-95 %, IC 1,27-3,46) était un facteur de risque pour les chiens, tandis que le sexe (OR : 3,40-95 %, IC 1,10-10,52) et la consommation d’aliments faits maison (OR : 8,49-95 % IC 2,48-29,05) l’étaient pour les chats. La consommation d’abats a été considérée comme un facteur de risque pour les deux espèces évaluées (OR : 2,74-95 %, IC 1,38-5,43 pour les chiens; OR : 7,66-95 %, IC 1,24-47,29 pour les chats). L’analyse spatiale a montré que les animaux séropositifs pour *T. gondii* étaient largement répartis dans la région métropolitaine de l’État de Rio de Janeiro, avec une concentration observée principalement dans les zones ouest et nord de la ville de Rio de Janeiro. Les résultats soulignent l’importance d’adopter des mesures prophylactiques pour contrôler la transmission de *T. gondii* chez les chiens et les chats domiciliés à Rio de Janeiro, contribuant positivement à la santé publique.

## Introduction

The protozoan *Toxoplasma gondii* is a globally distributed parasite capable of infecting homeothermic animals [19, 82]. Felids, including domestic cats, are definitive hosts because they are able to spread oocysts in the environment through feces, only in the days following the first infection [20, 53]. High humidity and temperatures above 30 °C favor the sporulation and maintenance of the oocysts in the environment [85]. However, high temperatures above 60 °C are able to inactivate *T. gondii* oocysts and cysts, the major parasite structures related to transmission for their hosts [22, 28]. For humans, the main transmission routes are the ingestion of raw or undercooked meat containing tissue cysts, and contaminated raw food and water with sporulated oocysts, or transplacental transmission [27]. In the urban context, domestic cats are considered the key host in the epidemiology of infection, as they are responsible for contamination of the environment shared with humans [17, 29]. Domestic dogs, as well as other mammals, including humans, and birds, are *T. gondii* intermediate hosts. The diet is an important factor for infection by *T. gondii* in domestic dogs and cats [25, 29]. The supply of leftovers, homemade food, and offal for these animals, especially dogs, may indicate possible sources of infection shared with humans.

In Brazil, there is high genetic diversity of *T. gondii* strains, including highly virulent clonal and atypical genotypes, isolated from different species of hosts [21, 23, 50, 59, 60, 72]. Among Brazilian people, up to 50% of elementary school children and 50–80% of women of child-bearing age have antibodies to *T. gondii* [23]. According to Shapiro et al. [71], the high prevalence of infection in this country is related to high environmental contamination by oocysts of the parasite. In addition, toxoplasmosis can determine severe clinical manifestations in adults and children from different regions of Brazil. According to Strang et al. [78], 48% of Brazilian children with congenital toxoplasmosis can develop retinochoroiditis, and 17% brain calcifications, among other changes. Regarding ocular toxoplasmosis, there is a wide variety of clinical outcomes of the disease in Brazilian individuals, which may be related to the high genetic diversity of the parasite in the country [32, 41].

High anti-*T. gondii* antibody frequencies have been reported in domestic dogs and cats from Cuba, Mexico, Panama, Iran, Argentina, Portugal, Australia, Colombia, and Chile [1, 10, 26, 30, 44, 57, 58, 63, 83, 86]. In Brazil, the frequency of *T. gondii* infection in Brazilian pet animal populations can vary from 9 to 70% in dogs and from 0 to 71% in cats [5, 9, 35, 47, 50, 68, 81]. *Toxoplasma gondii* seropositivity in pets can vary, not only between countries, but also between regions of the same country, and areas of the same city [20]. In general, domiciled animal populations or under the responsibility of owners have a lower frequency of anti-*T. gondii* antibodies compared to stray or shelter populations [4, 18, 47, 55, 77, 79]. Different risk factors for *T. gondii* infection have been identified in populations of domestic dogs and cats, including street access, contact with cats, and presence of rodents at home [2, 9, 12, 48, 64, 66, 67, 73]. Among these factors, the type of diet of these animals stands out. Strital et al. [79] identified an association between *T. gondii* seropositivity and offal consumption in dogs treated at a university veterinary hospital. According to Lucas et al. [45], diet and street access are important risk factors for cats. Coelho et al. [14] observed a positive association between diet and seropositivity in cats. In this study, all seropositive cats were fed homemade food, whereas cats fed exclusively with commercial food were not exposed to *T. gondii* [14]. In urban areas, where prey availability is low, the main source of infection by *T. gondii* for dogs and cats is the tissue cysts present in human food leftovers, available in garbage [49].

In 2013, according to the Brazilian Institute of Geography and Statistics (IBGE), the urban populations of domestic dogs and cats were 52.2 million and 22.1 million animals, respectively [39]. In the following six years, there was an increase in the number of households that have at least one pet in Brazil [39, 40]. This increase in human habitations with pets can narrow the contact between humans and animals, favoring zoonosis transmission. However, the introduction and diversity of commercial cat food on the Brazilian pet market may reduce *T. gondii* exposure in pet cats, because the heating and high temperatures applied to industrialized cat food eliminate viable tissue cysts [49]. In this context, there is a lack on seroepidemiological data on the frequency of anti-*T. gondii* antibodies that include pet animals from different socioeconomic classes, raised under different forms of ownership. In addition, there are no studies that identify the geolocation of domiciled animals exposed to *T. gondii* in large urban centers. Moreover, recently the Municipal Health Department of Rio de Janeiro city, by means of Resolution 3784 of August 21, 2018, mandates the notification of confirmed cases of zoonoses in animals, including toxoplasmosis [69].

Due to the importance of the role of dogs and cats in the epidemiology of toxoplasmosis and the scarcity of studies on this subject in Rio de Janeiro, we studied anti-*T. gondii* antibodies in domiciled animals, associated with the use of questionnaires to owners of low socio-economic classes to identify risk factors for pets. The identification of risk factors for pets allows the adoption of preventive measures to minimize the exposure of these animals to the protozoan. Considering that the different ownership profiles of pet owners directly reflect the food management of their animals, the present study aimed to assess the frequency of *T. gondii* infection, the spatial distribution of these animals, and risk factors for this infection. The study examined domestic dogs and cats attended at the Municipal Institute of Veterinary Medicine Jorge Vaitsman, Rio de Janeiro, Brazil, to carry out a targeted information and prevention campaign on the risks of *T. gondii* transmission in pets but also in humans.

## Materials and methods

### Ethical considerations

This research was approved by the Ethics Committee on the Use of Animals, IOC/Fiocruz, under license L-019/2017; by the Human Research Ethics Committee, IOC/Fiocruz project CAAE: 67408817.9.0000.5248, with approval number 2,054,938; by the Scientific Committee of the Secretariat for Surveillance, Sanitary Inspection and Zoonosis Control of Rio de Janeiro city under licenses 001/17 and 004/20.

### Animal population and study area

The study included domestic dogs and cats seen in the routine of the medical clinic of the Municipal Institute of Veterinary Medicine Jorge Vaitsman (IJV) whose owners agreed to participate in the research by signing a free and informed consent form. The IJV is located in the São Cristóvão neighborhood in the northern zone of Rio de Janeiro city and is considered a reference center in epidemiological surveillance and in the control of zoonoses of public health importance [80]. The Institute offers various veterinary services to the population of Rio de Janeiro, such as clinical care for animals, especially dogs and cats, surgical procedures, diagnostic tests, and vaccination either free of charge or at affordable prices.

Between August 2017 and January 2020, serum samples from 400 dogs and 272 cats were collected from animals sent for biological sample collection for routine, preoperative, and/or diagnostic confirmation tests. Clinically healthy and ill animals, males and females of different ages, and different breeds were included by means of convenience sampling.

### Blood collection and serological test

Blood samples were obtained through venipuncture of the cephalic, jugular, saphenolateral or femoral veins, with a maximum volume of 5 mL from healthy animals. In the case of young or clinically debilitated animals, a maximum volume of 3 mL or 6–7% of the animal’s weight was collected [43]. The samples were placed in tubes without anticoagulant, kept under refrigeration at 4 °C and transported to the Toxoplasmosis and other Protozoan Diseases Laboratory (LabTOXO), Oswaldo Cruz Institute/Fiocruz.

In LabTOXO, blood samples were centrifuged at 1000 ×*g* for 10 min to obtain the serum. Serum samples were aliquoted in 1.5 mL microtubes, identified, and stored at 20 °C until the serological test was performed. The serum samples were analyzed for antibodies to *T. gondii* by the indirect fluorescent antibody test IFAT, as described by Camargo [11]. The *T. gondii* tachyzoites RH strain, maintained in Swiss Webster mice, was used as an antigen. Positive and negative controls of each species, stored in LabTOXO, were used for each reaction. For the detection of IgG anti-*T. gondii* antibodies, commercial anti-Cat IgG produced in goats, BioRad^®^ and anti-Dog IgG produced in rabbit Sigma–Aldrich^®^ conjugates diluted in Evans Blue solution were used. The samples were considered positive when total fluorescence of the tachyzoite surface was observed, with titers equal to or greater than 1:16 in dogs and 1:64 in cats [5, 61].

### Epidemiological questionnaire

After the collection of blood samples, the animal owners were interviewed to answer a semi-structured epidemiological questionnaire composed of semi-open questions. This questionnaire included epidemiological variables related to general information about the animal’s lifestyle and management. To identify possible risk factors for *T. gondii* infection in these animals, the following variables were selected: sex [52, 86], age [5, 65], breed [6, 56], street access [9, 67], contact with cats [12, 66], presence of rodents [48], type of food (dry and wet commercial food, meat and embedded, homemade food, offal and other types of food, like fruits and vegetables) [14, 79] and water source for animals’ consumption [8].

### Statistical analysis and georeferencing

Serological and epidemiological data were analyzed using the Epi Info statistical program (version 7.2). Initially, an univariable exploratory analysis of the data was carried out for the selection of variables with *p* ≤ 0.2 using the chi-square or Fischer exact tests. Subsequently, the significant variables passed to a multivariable analysis using the multiple logistic regression with significant level of 5%. The existence of associated factors was estimated by multiple logistic regression and the strength of the association was estimated by odds ratio (OR) and their respective 95% confidence intervals. The animal’s place of residence was recovered using semi-structured questionnaires. Maps of the distribution of sampled domestic dogs and cats from different areas of Rio de Janeiro city and neighborhood cities from the metropolitan region of Rio de Janeiro state were constructed using ArcGis 10.1.

## Results

From the total number of animals evaluated, 34% of dogs (136/400) and 8.1% of cats (22/272) had IgG antibodies against *T. gondii*. Among dogs, the most frequent antibody titers were 1:16 (53.7%), followed by 1:64 (33.8%), 1:256 (11%) and 1:1024 (1.5%). The most frequent titer among felines was 1:64 (59.1%), followed by 1:256 (40.9%).

Owners of 321 (80.3%) dogs completely answered the questionnaire, while 36 (9%) partially answered. In relation to cats, 218 (80.1%) animals had their respective questionnaires completely answered by their owners, while 40 (14.7%) feline questionnaires were incompletely answered. Data from 43 (10.7%) dogs and 14 (5.2%) cats were not recovered, as their owners did not answer the questionnaires.

Regarding the geographic location of the dogs’ homes, 92.1% (315/342) lived in Rio de Janeiro city. The other dogs came from other cities in the metropolitan region of Rio de Janeiro state, with 2.6% (9/342) from São João de Meriti, 1.4% (5/342) from Nova Iguaçu, 0.9% (3/342) from São Gonçalo, 0.9% (3/342) from Duque de Caxias, 0.6% (2/342) from Belfort Roxo, 0.6% (2/342) from Mesquita, 0.3% (1/342) from Magé, 0.3% (1 /342) from Queimados, and 0.3% (1/342) from Itaboraí. Among the cats, 97.6% (241/247) were from Rio de Janeiro city. The other cats came from São Gonçalo 0.8% (2/247), Duque de Caxias 0.4% (1/247), Belford Roxo 0.4% (1/247), Niterói 0.4% (1/247), and Mesquita 0.4% (1/247). The addresses of 14.5% of dogs (58/400) and 9.2% of cats (25/272) were not available because their owners did not inform them when filling out the epidemiological questionnaire.

**Figure 1:**
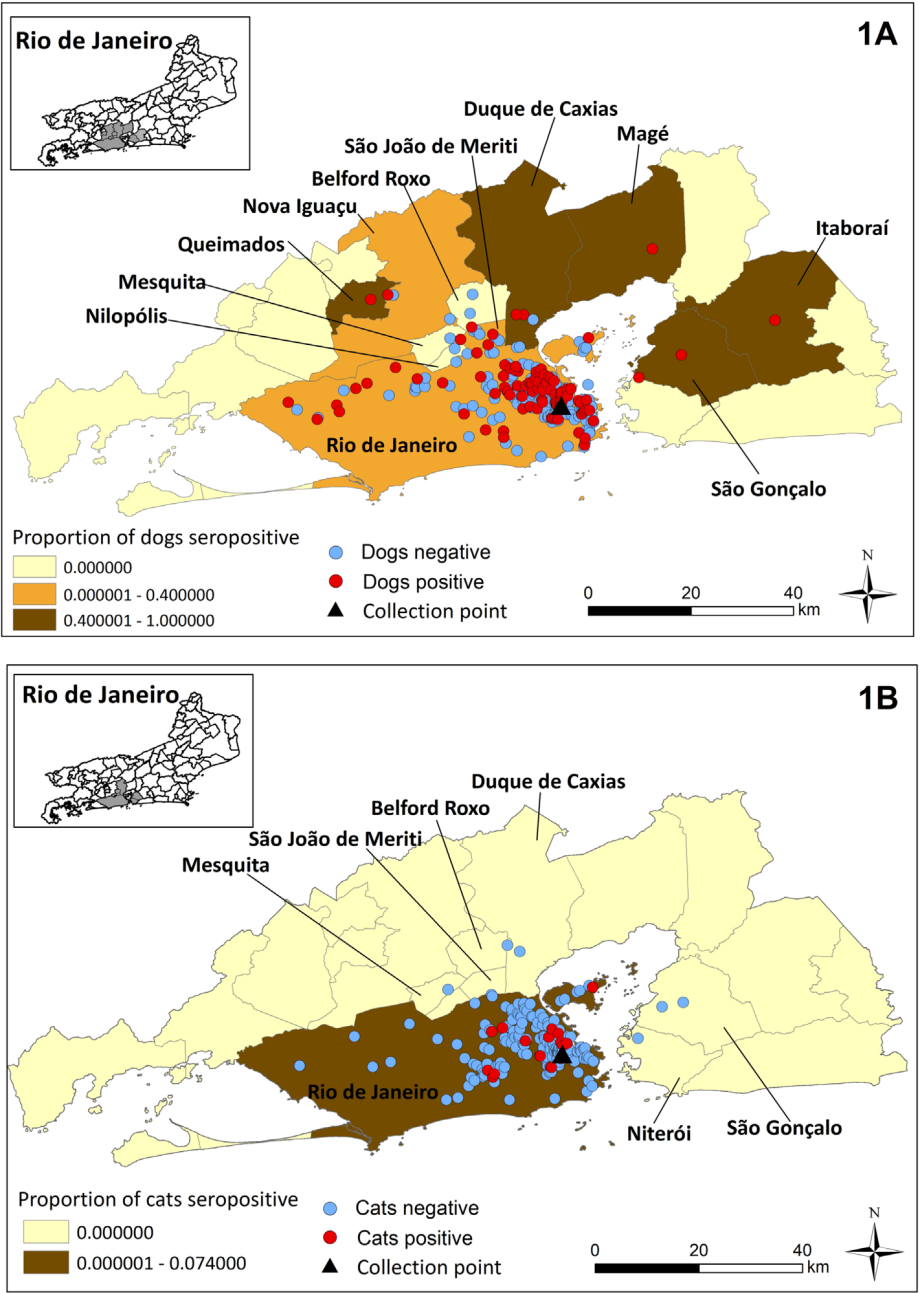
Spatial distribution of domiciled dogs (A) and cats (B) and proportion of *T. gondii-*seropositive animals by cities of the metropolitan region of Rio de Janeiro state.

**Figure 2:**
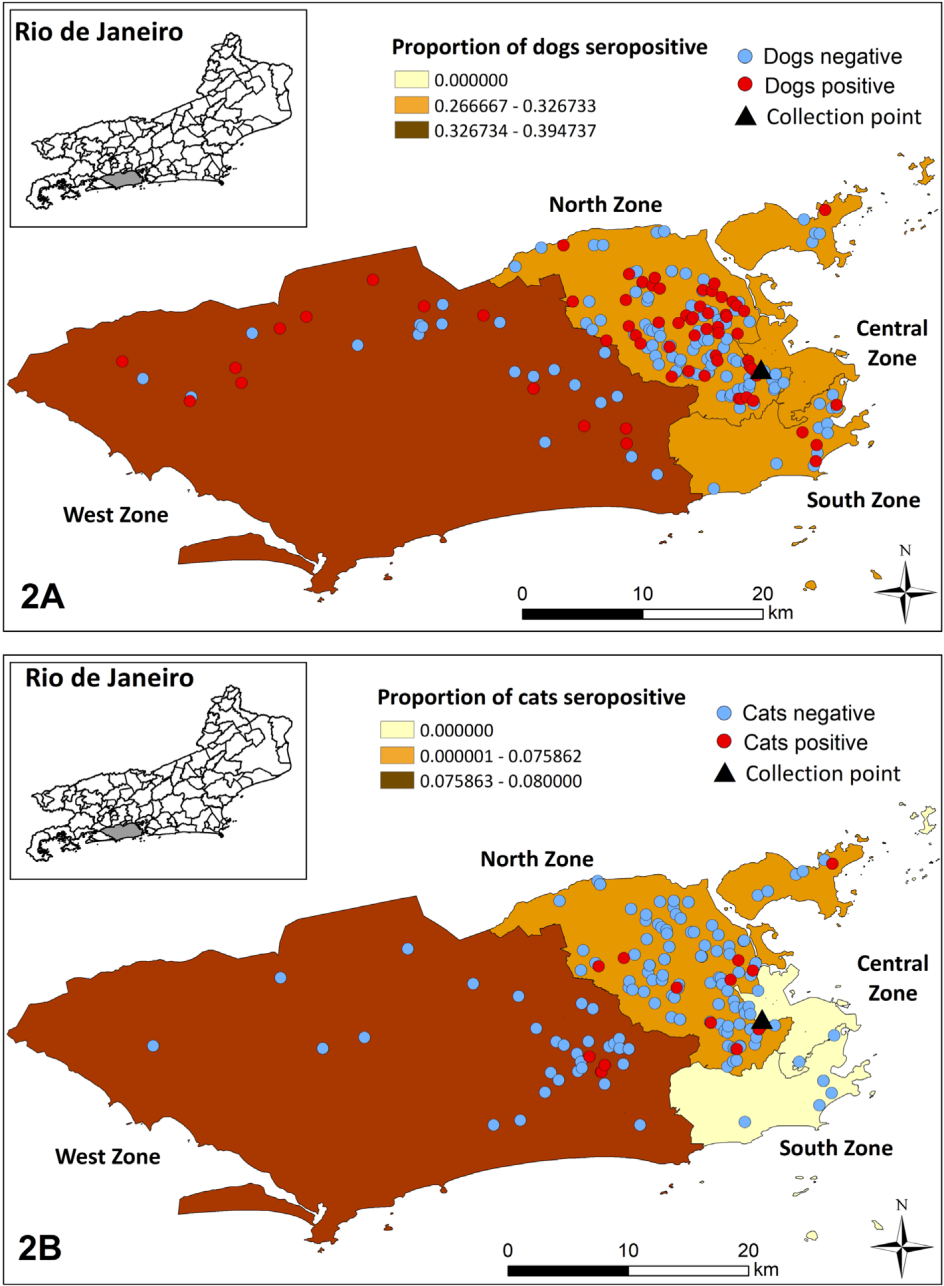
Spatial distribution of domiciled dogs (A) and cats (B) and proportion of *T. gondii-*seropositive animals by areas of Rio de Janeiro city.

As expected, the majority of *T. gondii* seropositive dogs, 89.6% (104/116) and all *T. gondii* seropositive cats, 100% (19/19) lived in Rio de Janeiro city, result directly observed by the concentration of geolocation points on the maps of the metropolitan region of Rio de Janeiro state. The other 12 *T. gondii-*seropositive dogs lived in São Gonçalo (3 dogs), São João de Meriti (3 dogs), Duque de Caxias (2 dogs), Itaboraí (1 dog), Magé (1 dog), Nova Iguaçú (1 dog), and Queimados (1 dog) (Figs. 1A and 1B). Although there was a higher concentration of *T. gondii-*seropositive animals in the northern area of Rio de Janeiro city (Figs. 2A and 2B), proportionally, the region of the city that showed the highest frequency of seropositive dogs was the west zone, 43.2% (16/37) followed by north zone, 33.8% (76/225), south zone, 29.4% (5/17), and central zone, 19.4% (7/36). In relation to cats, the region that presented the highest proportional frequency of seropositive animals was also the west zone, 8.9% (4/45), followed by north zone, 8.2% (12/146), central zone, 7.7% (3/39), and south zone, 0% (0/11). There was no association between the *T. gondii* seropositivity and the pets’ origin region in Rio de Janeiro city (Table 1).

**Table 1:** Frequencies of *T. gondii-*seropositive domestic dogs and cats by geographical region of Rio de Janeiro city treated at the Municipal Institute of Veterinary Medicine Jorge Vaitsman from August 2017 to January 2020.

Geographical area in Rio de Janeiro city	Dogs			Cats		
	*N*	Positive (%)	*p*-value	*N*	Positive (%)	*p*-value
Central zone	36	19.4	0.1787	39	7.7	0.7943
North zone	225	33.8		146	8.2	
South zone	17	29.4		11	0	
West zone	37	43.2		45	8.9	
Total	315			241		

The univariate data analysis revealed an association between the *T. gondii* seropositivity and the following variables: age, breed, and wet food, offal, and filtered water or other water sources for dogs (Table 2). In the final multiple regression model, breed and offal consumption were associated with *T. gondii* seropositivity (*p* < 0.05). Dogs without defined breed were twice as likely to be exposed to *T. gondii* (OR: 2.10–95% IC 1.27–3.46) compared to dogs with defined breed. Offal consumption increased twice (OR: 2.74–95% IC 1.38–5.43) the chance of infection by *T. gondii* in the studied dogs (Table 3).

**Table 2:** Univariable analysis (*p* ≤ 0.20) of the associated factors with *T. gondii* infection in domestic dogs and cats treated at Municipal Institute of Veterinary Medicine Jorge Vaitsman from August 2017 to January 2020.

Variables	Dogs			Cats		
	*N*	Positive (%)	*p*-value	*N*	Positive (%)	*p*-value
Sex						
Females	231	32.0	0.388^a^	157	5.7	0.096^a^,*
Males	169	36.7		115	11.3	
Age						
≤12 months^c^	70	24.3		94	5.3	
13–36 months	63	46.0	0.008^a^,*	60	5.0	1^b^
≥37 months	229	31.9	0.226^b^	78	9.0	0.382^b^
Breed						
Defined	191	25.1	0.001^a^,*	24	8.3	1^b^
Undefined	197	42.1		243	7.8	
Street access						
Yes	240	32.1	0.856^a^	80	12.5	0.062^a^,*
No	134	33.6		175	5.7	
Contact with cats						
Yes	93	38.7	0.283^a^	240	8.3	1^b^
No	294	32.0		3	0.0	
Presence of rodents						
Yes	109	32.1	0.838^a^	61	11.5	0.224^a^
No	236	33.9		181	6.6	
Type of food						
Dry food	367	33.2	0.308^a^	255	7.4	1^b^
Wet food	77	42.9	0.087^b^,*	114	8.8	0.460^a^
Meat and embedded	113	35.4	0.786^a^	46	13.0	0.108^a^,*
Homemade food	179	36.9	0.306^a^	36	22.2	0.0003^a^,*
Offal	43	53.5	0.007^b^,*	17	23.5	0.028^b^,*
Others	44	31.8	0.884^b^	4	0.0	1^b^
Water source						
Tap water	214	36.4	0.236^a^	149	8.7	0.391^a^
Filtered water	182	29.7	0.139^a^,*	121	6.6	0.592^a^
Other sources	2	100	0.113^b^,*	2	0.0	1^b^

a *X*² test.

b Fischer Exact test.

c Reference category.

* Selected for multivariate analysis.

**Table 3:** Final models of logistic regression analysis from variables statistically (*p* ≤ 0.05) associated factors with *T. gondii* infection in domestic dogs and cats treated at Municipal Institute of Veterinary Medicine Jorge Vaitsman from August 2017 to January 2020.

Species	Variable	Multiple logistic regression					
		Coefficient	Standard error	*P*-Wald	Degrees of freedom	*p*-value	Adjusted OR [95% CI]
Dogs	Age	0.1572	0.1552	1.0133	6	0.3109	1.1703 [0.8634–1.5862]
	Breed	0.7444	0.2539	2.9314	6	0.0034*	2.1051 [1.2798–3.4627]
	Wet food	0.4582	0.3009	1.5227	6	0.1278	1.5812 [0.8767–2.8518]
	Offal	1.0094	0.3487	2.8951	6	0.0038*	2.7440 [1.3855–5.4344]
	Filtered water	−0.0909	0.2511	−0.3620	6	0.7173	0.9131 [0.5582–1.4936]
	Other water sources	11.9799	310.0049	0.0386	6	0.9692	159513.8940 [0.0000 > 1.0E12]
Cats	Sex	1.2259	0.5752	2.1311	5	0.0331*	3.4071 [1.1035–10.5201]
	Street access	0.5349	0.5855	0.9135	5	0.3610	1.7072 [0.5419–5.3786]
	Meat and embedded	−1.2432	0.8846	−1.4054	5	0.1599	0.2884 [0.0509–1.6333]
	Homemade food	2.1390	0.6277	3.4077	5	0.0007*	8.4913 [2.4812–29.0591]
	Offal	2.0367	0.9285	2.1935	5	0.0283*	7.6652 [1.2422–47.2995]

* Statistically associated.

In cats, the selected variables in univariate analysis were sex, street access, meat and embedded, homemade food and offal consumption (Table 2). The final multiple regression model revealed an association between *T. gondii* seropositivity and sex, homemade food, and offal consumption (*p* < 0.05). Male cats were three times as likely to be exposed to *T. gondii* (OR: 3.40–95% IC 1.10–10.52) compared to females. Homemade food and offal consumption increased the chance of *T. gondii* exposure in the evaluated cats by eight (OR: 8.49–95% IC 2.48–29.05) and seven (OR: 7.66–95% IC 1.24–47.29) times, respectively (Table 3).

## Discussion

In this study, the frequency of seropositive dogs (34%) for *T. gondii* was higher than cats (8.1%). In other studies also carried out in Rio de Janeiro state, which included domiciled dogs and cats, seropositivity for anti-*T. gondii* were found to be higher for dogs (46.1%) and lower for cats (5.6–6.6%) [3, 4, 16]. Although these studies also included domiciled animals, Bastos et al. [4] and Cunha et al. [16] used indirect hemagglutination test (HAI) and enzyme linked immunosorbent assay (ELISA) as a serological test for the detection of antibodies anti-*T. gondii* in pets, respectively. Barros et al. [3], evaluated the presence of antibodies against *T. gondii*, also by IFAT, but only in domiciled cats with sporotrichosis treated at Laboratory of Clinical Research in Dermatozoonosis in Domestic Animals National Institute of Infectious Diseases (INI) / Fiocruz. In the present study, we chose to use IFAT for detection of antibodies against *T. gondii* in pet animals because this serological test is recommended for defining the true serological status of dogs and cats [46]. It is important to note that comparisons between these studies should be interpreted with caution, considering the use of different serological techniques, established cut-off points, and conditions for handling the animals. However, the serological results of the present study confirm those previously carried found in the same region, identifying similar seropositivity to those already reported in pet animals in Rio de Janeiro. In addition, unlike the previous studies, the present study included dog and cat populations analyzed simultaneously, in a socio-economically heterogeneous sample panel, as the sample collection was carried out in a Municipal Institution of notorious public health recognition.

The greater seropositivity evidenced in dogs compared to cats for *T. gondii* in Rio de Janeiro may be related to several factors, including the feeding behavior of the two animal species. This profile was also observed by Meireles et al. [49] in stray dogs and cats in São Paulo, Brazil. While dogs are less selective in their search for food, cats are strictly carnivorous animals; in other words, their diet consists exclusively of meat-based foods. Moreover, cats drink less water and eat smaller amount of food than dogs, which may reduce their exposure to *T. gondii* [49]. Therefore, dogs may be more likely to ingest one of the *T. gondii* evolutionary forms, including tissue cysts in raw or undercooked meat or oocysts in raw foods contaminated with feline feces, such as fruits and vegetables. The lower seropositivity in the cats tested than in the other studies may be related to the high proportion of cats fed commercial dry food in the population analyzed. Another factor that may be related to the higher frequency of infection in dogs than in cats is the habit of their owners walking in the streets with their dogs, a common practice in tropical regions, such as Rio de Janeiro state. While most dogs are taken out for a walk on the street and defecate, most cats are kept confined in the home environment. This restriction of the circulation environment may minimize the exposure of felines to the outdoor environment, possibly contaminated with *T. gondii* oocysts and inhabited by potential chronically infected intermediate hosts. It is important to highlight that the stray cat populations, resident in different areas of Rio de Janeiro city, are exposed to *T. gondii*, suggesting that these animals may be contributing to the contamination of the environment by oocysts in the study area [7, 51, 61].

The large number of seronegative cats observed in the cat population studied is of great importance, since if exposed to *T. gondii*, these animals will be able to spread large amounts of oocysts in the home or peridomiciliar environment, increasing the risk of infection by their owners and other animals. Therefore, preventive measures should be directed mainly to the seronegative cat population [24]. The occurrence of seropositive dogs can be considered a parameter of environmental contamination by the protozoan [6, 84]. According Boa Sorte et al. [6] and Ullmann et al. [84], the high prevalence of infected dogs in Brazil is due to the exposure of the dogs to waste and environmental contamination with *T. gondii* oocysts, showing the role of the dog as a sentinel animal for toxoplasmosis. Gao et al. [33] and Meireles et al. [49] indicates that canine toxoplasmosis might be an important epidemiological indicator of the risk of toxoplasmosis to humans. In addition to their role as a sentinel species, domestic dogs can mechanically carry *T. gondii* oocysts in their gastrointestinal tract through coprophagy after ingesting cat feces containing oocysts [31, 42]. Thus, the detection of *T. gondii* seropositive domiciled dogs in this study may indicate contamination of the environment shared with their owners.

In the present study, most seropositive dogs had low antibody titers detected by IFAT (53.7% at 1:16). This profile was also reported by Strital et al. [79] in dogs attended at the Veterinary School Hospital of the University of Mato Grosso (1:16–46%), by Brasil [9] in domiciled dogs attended at 34 veterinary clinics in Paraíba (1:16–35.1%), both in Brazil and by Hosseininejad et al. [38], in domiciled and stray dogs in Iran (1:16–42.7%). In relation to the seropositive cats detected in this study, the titration most frequently observed in IFAT was 1:64 (59.1%). This titration was also the most frequent in domiciled cats in Paraná and Acre, Brazil, and in domiciled cats in Iran, when the same technique was used [15, 36, 76]. In the Czech Republic, Sedlak and Bartova [70] reported high IgG titers in samples of domiciled dogs and cats submitted to IFAT of 1:10,240 and 1:81,920, respectively.

Despite the phylogenetic proximity of *T. gondii* and *Neospora caninum*, coccidia that have dogs and other canids as definitive hosts, the frequency of cross-reaction between antibodies against these protozoa is low in serum samples from dogs [37]. Thus, the establishment of the 1:16 titration as a cut-off point for serology in dogs evaluated in this study may have favored an increase in the sensitivity of the technique, that is, the detection of animals truly exposed to *T. gondii*. In contrast, the antigenic similarity between *T. gondii* and *Hammondia hammondi*, another Sarcocystidae coccidian that also has domestic cats as the definitive host, allows the occurrence of a cross-reaction between antibodies against these agents [34]. For this reason, the choice of 1:64 titration in cats included in the present study aimed to reduce the possibility of cross-reaction, in view of the more precise discrimination between felines truly exposed to *T. gondii*. It is worth mentioning that such differentiation is of great importance in public health, since *H. hammondi* has no potential for zoonotic transmission, such as *T. gondii*.

Although the sample size was not representative, the spatialization of sampled pets, especially dogs, showed that *T. gondii* can be widely disseminated, as was evidenced in dogs from 8 of the 19 cities of the metropolitan region of Rio de Janeiro state. In relation to the areas of Rio de Janeiro city, the plotting of the points referring to dogs indicated the circulation of the protozoan in all areas of the state capital, especially west zone. It is important to highlight that this region of Rio de Janeiro city concentrates neighborhoods with markedly different socio-economic profiles, including those with low social indicators, a scenario that may have favored the formation of seropositive animal clusters in this region. In this context, precarious animal handling with the supply of food leftovers, access to garbage, low sanitary conditions, the presence of synanthropic animals, and little concern by owners with animal health is included. For cats, spatial analysis indicated that oocysts of the parasite possibly contaminated the environment in all areas of Rio de Janeiro city, once there was no difference between frequencies of seropositive cats from different regions. The information generated from the spatial distribution of the animals, highlights the need to carry out actions and awareness campaigns for owners in all parts of the city, to mediate information about the control of *T. gondii* infection in these animals.

In this study, male cats were more exposed to *T. gondii* than females. However, Neves et al. [58] and Pereira et al. [61] found no association between sex and the presence of anti-*T. gondii* antibodies in other feline populations. According to Miró et al. [52] and Smith et al. [74], due to the explorative behavior of males, they end up being more prone to *T. gondii* infection than females. This exploratory behavior, possibly related to a higher energy requirement, may have contributed to the significantly higher frequency of seropositive male cats evaluated in this study. Undefined breed dogs had a higher frequency of anti-*T. gondii* antibodies than pure breed dogs. This profile was also evident in domiciled dogs in Tocantins and Mato Grosso, Brazil [6, 62]. This fact may be related to the difference in the types of care and sanitary management performed by their owners. In this sense, it is possible that owners of mixed breed dogs were investing little or no resources in the management of animals. Considering that dogs of different breeds are susceptible to *T. gondii* infection, the higher frequency of undefined breed dogs seropositive for *T. gondii* in this study may also be related to the eating habits of these animals.

This study confirmed that the supply of offal is an important risk factor for *T. gondii* infection in evaluated pets. Also, the consumption of homemade food was associated with greater exposure to the protozoan only in the feline population. The consumption of raw meat, offal and leftover homemade food was also associated with seropositivity for *T. gondii* in other populations of domiciled dogs and cats in Brazil and in other countries [10, 13, 54, 75, 79]. This result indicates that the exposure of domiciled dogs and cats to *T. gondii* occurs in the home environment through the supply of these foods by the owners. This behavior by some owners may favor the ingestion of viable tissue cysts present in the raw meat and offal offered to the animals. In addition, improper washing of raw food and cooking meat at low temperatures can be ineffective for inactivating infectious forms of the protozoan. The supply of these types of food added to the lack of knowledge about toxoplasmosis, as well as their transmission pathways and preventive measures by owners may have contributed to the exposure of dogs and cats to the parasite.

Given the above, the adoption of appropriate management practices, such the exclusive supply of industrialized food and the restriction of access of animals to places possibly contaminated by oocysts and/or cohabited by potential intermediate hosts can minimize the exposure of pet animals to *T. gondii*. This study confirms the importance of informing dog and cat owners not to feed raw food, especially offal, to their pets. The adoption of these prophylactic measures to control *T. gondii* infection in pet populations should be increasingly encouraged in public spaces, such as the Jorge Vaitsman Institute, where veterinarians work in clinical care, as well as in sensitizing owners to disseminate prophylactic information which indirectly contributes positively to public health.

## Conflict of interest

The authors declare that they have no conflict of interest.
